# Endogenous Panophthalmitis and Eye Enucleation Secondary to Methicillin-Resistant Staphylococcus aureus Bacteremia: A Rare Complication of Tunneled Dialysis Catheter Use

**DOI:** 10.7759/cureus.35107

**Published:** 2023-02-17

**Authors:** Joao Pedro T Batista, Zaid Hamarsha, Susie Q Lew

**Affiliations:** 1 School of Medicine, Universidade Federal de Minas Gerais, Belo Horizonte, BRA; 2 Critical Care Medicine, Tufts Medical Center, Boston, USA; 3 Medicine, George Washington University, Washington DC, USA

**Keywords:** enucleation, catheter-related bloodstream infection, hemodialysis, tunneled dialysis catheter, panophthalmitis

## Abstract

Catheter-related bloodstream infections are among the lethal complications of central venous catheter use. Patients with end-stage kidney disease use tunneled dialysis catheters (TDC) in the absence of arteriovenous access. We report a case of a patient using a TDC who developed panophthalmitis. This patient presented with painful and swollen eyes, fever, and chills. Positive methicillin-resistant *Staphylococcus aureus* (MRSA) blood cultures were thought to be secondary to a catheter-related bloodstream infection originating from his TDC. A maxillofacial computed tomography scan showed an enlarged, elongated, and proptotic left globe with suspected scleral irregularity suggestive of panophthalmitis. Despite TDC removal and systemic antibiotics, his left eye had to be enucleated. A new TDC was placed after treating the catheter-related bloodstream infection. He continued antibiotic therapy for a total of eight weeks. Panophthalmitis, a rare complication of catheter-related bloodstream infection among hemodialysis patients using a TDC, represents another reason to avoid TDC as hemodialysis access.

## Introduction

Among the most frequent and lethal complications from central venous catheters (CVCs) include catheter-related bloodstream infections (CRBIs). Bloodstream dissemination of the causative agent(s) can result in metastatic complications, with endocarditis, vertebral osteomyelitis, and spinal epidural abscess among the most common complications [1]. The present case reports an extremely rare event of panophthalmitis secondary to methicillin-resistant *Staphylococcus aureus* (MRSA) bacteremia in a catheter-dependent hemodialysis patient. This case was accepted by the 2022 National Kidney Foundation, National Capital Area Meeting for poster presentation on June 8, 2022.

## Case presentation

A man in his 30s, with a past medical history of hypertension, type 1 diabetes mellitus, heart failure with reduced ejection fraction and poor cognition secondary to a prior anoxic brain injury, presented with a swollen left eye and suspected MRSA bacteremia. His poorly controlled type 1 diabetes mellitus (DM) (hemoglobin A1C level at 9.3% at admission), resulted in end-stage kidney disease (ESKD). He started hemodialysis (HD) at the beginning of 2021. The patient also developed bilateral loss of visual acuity towards the end of the year secondary to diabetic retinopathy and non-ischemic cardiomyopathy.

The patient and his mother had reservations about his medical stability to undergo surgery, preventing him from getting an arteriovenous fistula (AVF) once he was initiated on hemodialysis. He used a tunneled dialysis catheter (TDC) for hemodialysis for approximately 12 months. He had several hospitalizations during his first year of dialysis for multiple episodes of bloodstream infections associated with his TDC. One of these admissions, in May 2021, was reportedly associated with MRSA bacteremia that required TDC removal and replacement. The patient denied any vision decline or eye symptoms during this prior episode.

On the present occasion, the patient was initially admitted to an outside hospital in January 2022, complaining of pain in his right eye, a severely swollen left eye, fever, and chills that have been present for the last four to five weeks. He had suffered from left eye swelling since the end of November 2021. Due to his poor cognition secondary to an anoxic brain injury that resulted from a prior cardiac arrest, the patient was unable to specify details. He stated he had not seen any doctors for these symptoms before, and when asked why he did not seek help, the patient responded, “I don’t know.” He was residing in a nursing home at the onset of symptoms. His symptoms progressively worsened, which resulted in his hospitalization at the beginning of 2022. The patient reported that his vision allowed him to count fingers two to three weeks prior to hospitalization. When he sought medical care, he could only see light from his right eye. At the outside hospital, blood cultures grew MRSA, which was thought to be secondary to a CRBI from his TDC and endophthalmitis was suspected. The patient received intravenous daptomycin and ceftaroline antibiotics. The TDC was replaced by a non-tunneled temporary catheter in his right internal jugular (RIJ) vein. A transesophageal echocardiogram (TEE) revealed left ventricular systolic dysfunction with decreased ejection fraction, and no valvular vegetations were visualized. A computed tomography (CT) scan showed copious amounts of soft tissue swelling and inferior displacement of the left globe (image not available). Since the outside hospital could not send his complete record, we hypothesize the patient was started on two classes of antibiotics given an unfavourable antibiotic susceptibility pattern and reportedly a past medical history of MRSA bacteremia in 2021. 

**Figure 1:**
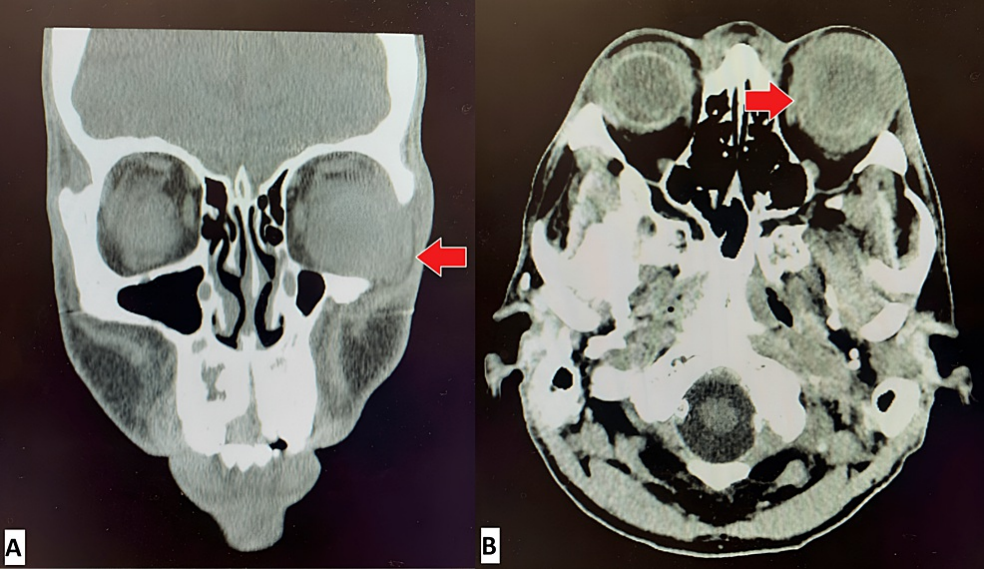
Patient’s head CT scan showing more left eye findings than the right Panel A: Head CT scan without contrast coronal view.  The arrow points to the left globe, showing an elongated and proptotic globe with scleral irregularity/discontinuity. The swelling extends into the post septal and intraconal space, with increased density within the posterior compartment and posterior aspect of the vitreous. Similar appearing, less severe findings are seen involving the right globe with circumferential swelling extending postseptally/intraconally with scleral irregularity and increased density within the posterior compartment. Panel B: Head CT scan without contrast axial view. The arrow points to the left globe, depicting left greater than right periorbital soft tissue swelling with fluid collections surrounding the globes more anteriorly. There is no evidence of thrombosis or superior ophthalmic veins or cavernous sinus thrombosis in this study.

Three weeks after admission to the outside hospital, he was transferred to our hospital for an ophthalmology consultation to evaluate a persistent swollen left eye. A second set of blood cultures collected at the outside hospital just prior to transfer was reported to be negative at the time of transfer. On admission, the patient was alert, afebrile, and without leukocytosis. His physical examination revealed bilateral eyelid swelling, worse on the left side, with drainage of purulent secretion. He did not have any heart murmur or back tenderness. The temporary catheter was intact and in place, with no infectious signs. A new CT maxillofacial without contrast (Figure 1) revealed an enlarged and proptotic left globe with scleral irregularity and swelling extending into the post-septal and intraconal space. Overall, these findings were concerning for infectious panophthalmitis.

He was switched from his initial antibiotic treatment of daptomycin and ceftaroline to piperacillin-tazobactam when admitted to our hospital, aiming for broader coverage. Four days later, when blood cultures did not result in any organism, piperacillin-tazobactam was replaced by daptomycin targeting MRSA specifically since his initial blood cultures were positive for the organism. Daptomycin was discontinued a few days later in favor of starting vancomycin, given that it would be easier to dose with HD. He continued antibiotic therapy for a total of eight weeks. His inflammatory markers trended down since the beginning of the treatment, with white blood cell count varying from a maximum of 17 x 10^9^ /L three days after admission and returning to a normal range after a few days of systemic antibiotic treatment initiation. C-reactive protein also significantly returned to the normal range after treatment initiation and clearing of blood cultures and maintained within normal levels until discharge. The pain was well controlled with acetaminophen, and non-steroidal anti-inflammatory agents were not administered.

Ophthalmology recommended left eye intervention with enucleation and debridement. Orbit fluid analysis from hospital day four revealed many polymorphonuclear leukocytes, a few gram-positive cocci, and fluid cultured MRSA. The enucleation of the left eye was performed one day later.

The MRSA bloodstream infection was diagnosed by the first blood cultures obtained at his external hospitalization and the right eye infection were successfully treated with antibiotic therapy. A new TDC replaced the non-tunneled dialysis catheter. He was discharged to a skilled nursing facility, with a follow-up appointment scheduled with the ophthalmologist, and he continued HD via a TDC. Better blood sugar control during hospitalization significantly reduced his hemoglobin A1C levels from 9.3% at admission to 7.7% at discharge. He also had plans to see a vascular surgeon to create an AV fistula. Unfortunately, the patient died from complications of pulmonary emboli one month after discharge.

## Discussion

Panophthalmitis represents a severe form of endophthalmitis that extends to the three coats of the eye and the adjacent tissue of the orbit. This rare condition presents with nonspecific findings, including edema/erythema of the eyelids, proptosis, limitation of extraocular movements, and vision loss [2]. Panophthalmitis most likely occurs secondary to exogenous etiologies, such as trauma to the eye and post-surgical complications [2]. In contrast, endogenous panophthalmitis results from infection from a secondary focus spreading into the globe. Risk factors include DM, urinary tract infection, recent hospitalizations, immunosuppression, intravenous drug abuse, and indwelling catheters [3]. The most common causative agents include *Staphylococcus* or *Pseudomonas* [4].

Panophthalmitis diagnosis relies primarily on clinical findings suggestive of eye inflammation. Frequently, nonspecific symptoms might delay the condition’s diagnosis, resulting in worse outcomes. Thus, physicians should be aware of and consider endogenous endophthalmitis/panophthalmitis among the differential diagnosis in symptomatic patients with risk factors. Microbiological techniques for staining and culture specimens from the eye should be obtained to identify the causative agent; however, it should not delay empiric antibiotic treatment initiation [5]. Imaging studies help differentiate panophthalmitis from endophthalmitis since the former has extensive eye tissue involvement; however, radiologic studies are not mandatory. An ultrasound of the eye provides additional information when the examiner cannot obtain clear visualization of the vitreous, showing increased echogenicity of the vitreous [6]. 

In the presence of symptoms suggestive of infection in a catheter-dependent hemodialysis patient, the presumed diagnosis should be catheter-related bacteremia [7]. However, other accesses with foreign material and non-access-related infections should also be considered. Since this patient did not have any other accesses that could serve as a primary source for his infection, his TDC was considered the primary source. Similarly, he did not have other symptomatic infectious foci. 

Given the patient’s baseline vision impairment, it was difficult in the beginning to discern whether further compromise of his vision was due to infection involving the globe or cellulitis in the adjacent tissue. His baseline loss of visual acuity may explain why ophthalmology was not initially consulted at the outside hospital. The diagnosis of panophthalmitis was established based on physical findings suggestive of orbital cellulitis and the CT findings that confirmed an extensive inflammation within the eye and in the orbital soft tissue. A plausible scenario suggests that a previous or current episode of MRSA bloodstream infection secondary to the TDC seeded the orbit resulting in panophthalmitis. 

Treatment of panophthalmitis can be divided into systemic or topical antibiotic therapy that crosses the blood-eye barrier. Broad-spectrum antibiotics should be combined with intravitreal injections to address the underlying systemic infection. Empirical intravitreal antibiotics should cover gram-positive and gram-negative organisms when the etiology remains unknown, including vancomycin and ceftazidime [5]. Both endophthalmitis and panophthalmitis have poor prognoses and can result in complete vision loss [8]. Vitrectomy helps correct persistent inflammation, while eye evisceration stays reserved for cases where antibiotics fail to control infection or those with severe tissue destruction [9].

The management of TDC-associated bacteremia consists of systemic antibiotics and prompt removal of the infected device since better outcomes are demonstrated with immediate removal compared to no removal and observed poor response to antibiotic therapy alone [10-11]. When chosen not to be removed, an alternative approach consists of using an antibiotic lock, which involves instilling a high concentration of antibiotic into each lumen of the TDC at the end of each dialysis session for the duration of systemic therapy [10]. The empirical treatment should provide coverage for gram-positive and gram-negative bacteria, including methicillin-resistant staphylococcal species since a substantial proportion of staphylococcal infections in dialysis patients is methicillin-resistant [12]. Therefore, vancomycin and aminoglycosides or third-generation cephalosporin should be used to cover gram-positive and gram-negative bacteria, respectively. The duration of treatment should be three weeks for uncomplicated bacteremia and six to eight weeks for patients with metastatic infection [10]. Left eye enucleation was indicated when panophthalmitis failed to respond to antibiotic therapy. Right eye ophthalmitis was treated with intravitreal moxifloxacin. Additional topical treatment consisted of dorzolamide-timolol, brimonidine, latanoprost, and atropine.

This patient’s antibiotic regimen included a systemic antibiotic treatment regimen of daptomycin and ceftaroline at the outside hospital and piperacillin-tazobactam at our hospital until repeat culture results were available. He was restarted on daptomycin per the infectious disease consult’s recommendation. Daptomycin was then discontinued in favor of starting vancomycin, considering it would be easier to dose with hemodialysis. Daptomycin replaced vancomycin when ideal serum vancomycin levels could not be achieved and continued for additional four weeks after his enucleation surgery, for a total time of antibiotic therapy of eight weeks. 

In this case report, the TDC was the source of the infection that spread hematogenously to reach the eye. A TDC is a suboptimal dialysis access option for patients with ESKD [13]. Patients rely on the immediate use of CVCs mainly as a bridge to future AVF creation or graft placement. It can also be a temporary option when permanent access fails. Typical disadvantages of dialysis catheters when compared to AVFs/grafts include decreased blood flow rate leading to decreased dialysis efficiency and increased risk of complications, including thrombosis, catheter dysfunction, and CRBIs [14].

A critical limitation in the management of this patient was the inability to verify if any catheter tip culture was performed at the outside hospital, essential information to confirm the TDC as the primary source of the bloodstream infection. This case represents the first publication reporting the incidence of panophthalmitis as a complication from CRBI in a dialysis patient. A few cases of endophthalmitis as a complication of bacteremia related to a TDC have been reported [4,15-17]. In three of the cases, the outcome was poor with eye evisceration [15-16] or vitrectomy [17]. The one report that did not result in eye evisceration also had a poor outcome, with significant impairment of the patient’s vision [4].

Additional limitations in the management likely contributed to the evolution of this patient’s condition to a severe presentation of panophthalmitis. His uncontrolled diabetes can weaken the immune response, allowing for an aggressive bacterial infection spread. Additionally, diabetic retinopathy could be considered a confounding factor in the initial presentation, preventing the primary team from better diagnosing any eye involvement and the patient himself from noticing a worsening in his visual function. This confounding factor resulted in a delayed diagnosis and treatment, requiring evisceration of the eye. Also, seeking medical evaluation two months after the beginning of symptoms likely contributed to the worse outcome. Strengths in managing this complicated case can also be noted. The prompt removal of the infected TDC to address the infectious source was wisely executed, and the empiric antibiotic treatment to treat the bloodstream infection was also timely introduced. The decision to proceed with left eye enucleation and left orbit debridement was necessary, given the failure of antibiotic treatment after three weeks. However, the right eye infection was successfully treated.

The patient’s refusal of AVF creation exposed him to TDC-related complications. International guidelines strongly recommend AVFs and discourage the use of CVCs for long-term access [18-19], emphasizing their use as a temporary measure. In a Canadian observational study [19], including one thousand patients, approximately one-third of hemodialysis individuals who used TDCs for one to two years experienced complications. Bacteremia, the most common cause of hospitalizations, occurred in nine percent of patients in one year. Compared to AVFs, long-term TDCs encounter a five to 10-fold increased risk of severe infection, a threefold increased risk of death, and increased hospitalization [20].

## Conclusions

This case report emphasizes a severe complication resulting from the long-term use of a TDC. TDCs should be employed as short or intermediate-term options for dialysis, acting as a bridge for AVF placement. Panophthalmitis, a rare complication arising from CRBIs, should be considered among the differential diagnosis in a patient with risk factors and suggestive symptoms on the ophthalmological examination since a delayed diagnosis is associated with poorer outcomes. TDC-related bacteremia treatment centers on antibiotic therapy and catheter removal. Treatment starts with broad-spectrum antibiotics, followed by antibiotics based on organism sensitivity results. Finally, the antibiotic choice is further refined to those that can be conveniently administered during dialysis in the outpatient dialysis unit for prolonged treatment of three weeks for uncomplicated bacteremia and up to eight weeks for metastatic infection.

Limitations in the management likely contributed to the evolution of this patient’s condition to a severe presentation of panophthalmitis. Diabetic retinopathy could be considered a confounding factor and seeking medical evaluation two months after the beginning of symptoms likely contributed to the worse outcome. The decision on the left eye enucleation and debridement of the left orbit was necessary, given the failure of antibiotic treatment after three weeks. However, the right eye infection was successfully treated.
